# A Comparison of Transcanalicular, Endonasal, and External Dacryocystorhinostomy in Functional Epiphora: A Minimum Two-Year Follow-Up Study

**DOI:** 10.1155/2022/3996854

**Published:** 2022-03-23

**Authors:** Can Ozturker, Bayasgalan Purevdorj, Gamze Ozturk Karabulut, Gamal Seif, Korhan Fazil, Yasser Anwar Khan, Pelin Kaynak

**Affiliations:** ^1^Department of Ophthalmic Plastic and Reconstructive Surgery, Istanbul Beyoglu Eye Research and Training Hospital, Bereketzade Cami Sk. No. 2, Beyoglu, Istanbul 34421, Turkey; ^2^Division of Ophthalmology, Department of Surgery, Faculty of Health Sciences, McMaster University, 1280 Main Street West, Hamilton, ON L8S 4K1, Canada; ^3^Department of Ophthalmology, Mongolian National University of Medical Sciences, Jamyan St 3, Ulaanbaatar 14210, Mongolia

## Abstract

**Purpose:**

To compare the outcomes of transcanalicular diode laser-assisted dacryocystorhinostomy (TCL-DCR), nonendoscopic endonasal dacryocystorhinostomy (NEN-DCR), and external dacryocystorhinostomy (EXT-DCR) as first-line treatments for functional epiphora.

**Methods:**

This multicenter, retrospective, case-control study included 135 eyes of 135 patients with functional epiphora (86 females and 49 males). Functional epiphora was diagnosed based on a patent lacrimal system with a delay in the fluorescein dye disappearance test (FDDT) or dacryoscintigraphy (DSG) and no ocular surface or eyelid abnormalities. The patients were treated with TCL-DCR (2008–2011) or Ext-DCR (2005–2008, 2011–2017) at Beyoglu Eye Research Hospital (Istanbul, Turkey) and NEN-DCR at Carrot Eye Surgery Clinic affiliated with the McMaster University (Hamilton, ON, Canada) (2010–2016). Success was defined as the absence of epiphora and the normalization of an earlier delayed FDDT after surgery.

**Results:**

The TCL-DCR, NEN-DCR, and EXT-DCR groups consisted of 38, 47, and 50 eyes with 25.9, 44.2, and 45.9 months of follow-up. The success rate for TCL-DCR was 65.8%, 70.2% for NEN-DCR, and 84.0% for EXT-DCR. During the follow-up period, 13.2% of TCL-DCR cases and 6.4% of NEN-DCR cases developed an anatomic obstruction of the lacrimal system.

**Conclusion:**

The EXT-DCR group had a higher success rate in the management of functional epiphora than the NEN-DCR and TCL-DCR groups and was significantly safer in terms of an iatrogenic anatomic block of the lacrimal system.

## 1. Introduction

Epiphora is the most common symptom of an anatomic obstruction of the lacrimal drainage system, which can occur at the level of the puncta, canaliculi, lacrimal sac, or nasolacrimal duct as a result of chronic inflammations, recurrent infections, or drug toxicity. Depending on the level of obstruction, treatment aims to recanalize or bypass the obstructed segment [1–5].

In the absence of dry eye, reflex tearing, or an abnormality of the eyelids, the presence of epiphora in the context of a patent lacrimal drainage system is commonly referred as “functional epiphora” or “functional nasolacrimal duct obstruction.” Perry J.D. introduced the term “dysfunctional epiphora,” emphasizing that a delay in lacrimal transit time indicates a defective lacrimal apparatus [6]. The lacrimal apparatus has three dynamic components: tear secretion, tear film evaporation, and lacrimal clearance [7]. An imbalance between these secreting and clearing components leads to functional epiphora.

The treatment options for functional epiphora generally aim to improve the lacrimal pump and lower lacrimal resistance. However, there is no consensus about the algorithm that needs to be followed [8–10]. If the lacrimal pump is already intact and does not require reinforcement, dacryocystorhinostomy (DCR) with bicanalicular silicone intubation is one of the treatment options for lowering proximal and distal drainage system resistance in a single procedure by connecting the lacrimal sac to the nasal cavity and dilating the canaliculi [11–14].

Despite its high success rate, external dacryocystorhinostomy (EXT-DCR) may leave a facial scar. Probable pre and postoperative bleeding, as well as longer procedure and recovery times, may occasionally become additional concerns. The hunt for an ideal DCR technique that can be performed quickly and easily with a high success rate and without leaving a scar still continues [15].

Eloy et al. first reported the use of diode laser for transcanalicular laser-assisted dacryocystorhinostomy in 2000, and it has since been studied by various researchers in order to observe the outcome and find ways to improve the results while minimizing complications [15–20]. According to reports, the advantages of diode lasers include their ability to deliver a sufficiently powerful laser beam via a relatively narrow optical fiber with less collateral heating and less residual thermal damage to the target tissue [20]. Other advantages of diode laser devices include portability, ease of use, and low maintenance costs.

This study was designed to compare the results of three different dacryocystorhinostomy techniques, transcanalicular diode laser-assisted dacryocystorhinostomy (TCL-DCR), nonendoscopic endonasal dacryocystorhinostomy (NEN-DCR), and EXT-DCR combined with bicanalicular silicone intubation, for the treatment of functional epiphora. It is the first study in the literature to report the results of TCL-DCR in functional epiphora and compare it with NEN-DCR and EXT-DCR.

## 2. Methods

This study was conducted retrospectively by working on the data of 135 patients with functional epiphora who were operated at the Ophthalmic Plastic and Reconstructive Surgery Departments of Beyoglu Eye Research Hospital (Istanbul, Turkey) and Carrot Eye Surgery Clinic affiliated with the McMaster University (Hamilton, ON, Canada) between April 2005 and March 2017. Data collection was completed in December 2020 after a mean follow-up of 39.7 months (6–97). All procedures performed in this study, involving human participants, were in accordance with the 1964 Helsinki Declaration and its later amendments or comparable ethical standards. Informed consents were obtained from all individual participants included in the study, and ethics committee approval was received for this study from the Ethics Committee of Istanbul University, Istanbul Faculty of Medicine (number: 12.07.2021–319162).

All patients with a history of tearing underwent full clinical and ophthalmic examinations. In case of a patent lacrimal irrigation, a fluorescein dye disappearance test (FDDT) or dacryoscintigraphy (DSG) was performed to reveal a delay in the lacrimal transit time and diagnose functional epiphora. The exclusion criteria were age under 18 years, dry eye (tear break up time ≤10 seconds), ocular surface disorders, eyelid malposition or laxity, orbicularis weakness, partial block or stenosis of the lacrimal drainage, history of previous nasal, lacrimal, or ophthalmic surgeries, and a postoperative follow-up time less than six months. In bilateral cases, only the right eye was included in the study to avoid any bias related to each patient's wound healing characteristics.

**Figure 1:**
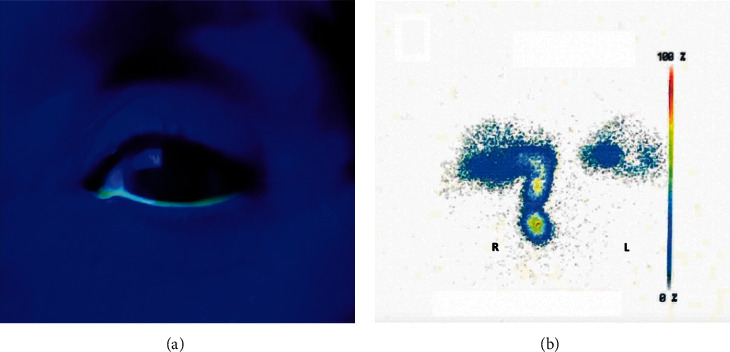
Delay of lacrimal transit on the left eye after 5 minutes demonstrated with fluorescein dye disappearance test (FDDT) (a) and dacryoscintigraphy (b).

Lacrimal syringing was performed by irrigating through the upper and lower puncta with a lacrimal cannula. For the FDDT, the dye was administered to the inferior fornix with one single drop of an ophthalmic antibiotic on a fluorescein strip and evaluated after 5 minutes (Figure 1(a)). Lacrimal scintigraphy was performed with 99 m Tc-DTPA in a regular fashion, and lack of activity in the nasal cavity after 5 minutes was considered as dysfunction of lacrimal drainage (Figure 1(b)).

All the patients assigned for surgery were preoperatively evaluated for any intranasal pathology that might narrow the inferior meatus and cause partial obstruction. The nasal examination was either done by the authors themselves or by an ear, nose, and throat specialist upon referral.

The surgeries were performed by skilled surgeons (YAK, PK, and CO). Prior to 2008, all DCRs at Beyoglu Eye Research Hospital were performed primarily through the external route. TCL-DCR became the most commonly preferred technique by patients between 2008 and 2011, after the laser device became available. TCL-DCR surgeries were discontinued after 2011 due to unsatisfactory results, and EXT-DCR became the primary route of treatment once more. As a result, cases meeting the inclusion criteria underwent EXT-DCR (PK and CO) from 2005 to 2008 and 2011 to 2017 and TCL-DCR (PK and CO) from 2008 to 2011. NEN-DCR was always the preferred DCR technique at Carrot Eye Surgery Clinic, and patients who met the inclusion criteria and were operated on (YAK and CO) between 2010 and 2016 were enrolled in the study group.

**Figure 2:**
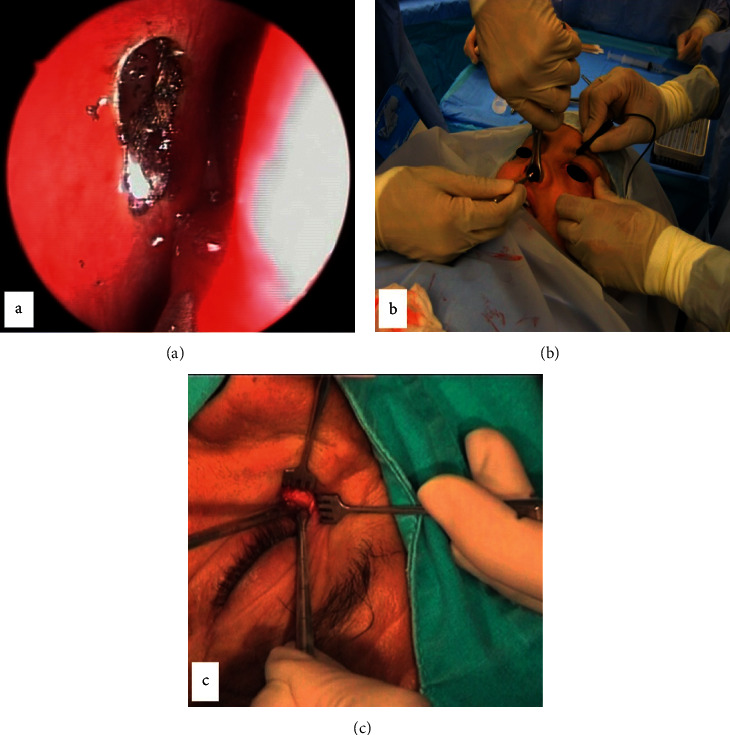
Intraoperative images from three different types of DCR surgeries: TCL-DCR (a), NEN-DCR (b), and EXT-DCR (c).

The surgical procedures were performed as standard techniques. A 980 nm solid state diode laser (Multidiode S30 OFT from INTERMEDIC, Spain) with a 600 m silica-silica polyamide laser fiber optic was used for TCL-DCR. Laser settings were adjusted between 8 and 12 W power range, 350–500 ms pulse time, and 350–500 ms pause duration between pulses. Topical mitomycin-C (MMC) (0.2 mg/ml) was applied for 2 minutes on the rhinostomy site in TCL-DCR cases routinely (Figure 2(a)) (See supplemental video, https://drive.google.com/file/d/1PZwKj7y5zQCmIhgv8z7l_ETmDkAni0xG/view?usp=sharing, which demonstrates the important steps of TCL-DCR surgery).

The surgery for NEN-DCR necessitated the use of surgical loupes and headlights. To transilluminate the lacrimal sac, a 20 G disposable vitrectomy light pipe was inserted through the upper canaliculus into the lacrimal sac. An elliptical mucosal flap was elevated and removed from the lacrimal bone, and osteotomy was created to completely expose the medial wall of the lacrimal sac. The lacrimal sac was vertically incised to create anterior and posterior flaps, which were then completely removed (Figure 2(b)).

The EXT-DCR procedures were performed through a 7-8 mm long nasojugal skin incision. Only anterior flaps were created and sutured together. After tying the knot, the sutures were passed through the periosteum and orbicularis muscle to reconstruct the lacrimal diaphragm and attach it to the mucosal flaps (Figure 2(c)).

All the EXT-DCRs were performed under sterile conditions, but only a clean setup was preferred for NEN-DCR and TCL-DCR procedures. Crawford bicanalicular silicone stents were placed and tied using a square knot in all cases.

Postoperative treatment consisted of oral antibiotics for the postoperative first week, intranasal steroid sprays and steroid eye drops q.i.d, for 6 weeks, and antibiotic eye drops q.i.d. for 2 weeks. In addition, for patients who underwent EXT-DCR, a topical antibiotic ointment was prescribed to be applied to the skin incision t.i.d. for 2 weeks to keep it clean and moist. After 2 weeks, it was replaced with a steroid ointment b.i.d. for another 2–4 weeks.

Postoperative follow-up visits were scheduled for the first day; first week; first, second, third, sixth months; and first year. Patients were asked to return after the first year of follow-up if they experienced any recurrent symptoms. The final status of the epiphora was reviewed for this study by calling the patients.

Patients were asked verbally about tearing of their eyes at each visit and underwent FDDT and lacrimal irrigation. The skin sutures in EXT-DCR cases were removed one week after surgery. Stent removal was planned for the postoperative second month. A “successful outcome” was defined as the absence of epiphora and normalization of an earlier delayed FDDT following the surgical procedure. In contrast, continuing epiphora with a delayed FDDT was described as a “failure.” A nonpatent lacrimal irrigation system was accepted as an “anatomic block” in the unsuccessful DCRs.

### 2.1. Statistical Analysis

The mean age and the mean follow-up time in the three groups were compared using one-way analysis of variance (ANOVA). The effect of age on success was evaluated using binary logistic regression analysis. The sex distribution, the effect of early stent removal on success, and the results of the three different surgical techniques were analyzed using the chi-square test. Statistical analysis was performed using the IBM SPSS Statistics 21.0 software (IBM Corp. Armonk, NY, U.S.A.), and *P* values <0.05 were considered statistically significant.

## 3. Results

This study included 135 patients (86 females and 49 males) divided into three groups based on the type of surgery. The TCL-DCR, NEN-DCR, and EXT-DCR groups consisted of 38, 47, and 50 eyes, respectively. The variance in gender distribution among the groups was significant (*p*=0.04) without any effect on success in any group (*p*=0.91, *p*=0.53, and *p*=0.72) (Table 1).

The mean age was higher in the NEN-DCR group (64.1 years) compared with TCL-DCR (49.3 years) and EXT-DCR groups (56.5 years). The mean follow-up times were 44.2, 25.9, and 45.9 months, respectively. The differences in mean ages and follow-up times were statistically significant (*p* < 0.01 and *p* < 0.01) (Table 1). There was no significant effect of age on success in any group (*p*=0.16, *p*=0.91, and *p*=0.22).

The mean time of stent removal was 2.3 months in total. In 47 cases (34.8%), the stents had to be removed earlier than planned due to stent prolapse or loss. These patients were only observed without secondary intubation. Stent removal earlier than two months was not associated with surgical failure in any group (*p*=0.54, *p*=0.17, and *p*=0.71) (Table 1).

All surgeries were performed uneventfully, except one TCL-DCR procedure where the lower canaliculus was injured related to an inadvertent laser shot in the canaliculus. The silicone stent prolapsed on the sixth postoperative week and had to be removed prematurely. After two years of follow-up, the patient had no stricture of the canaliculi and no recurrent epiphora. Postoperatively, one TCL-DCR case had slitting of the lower canaliculus, one EXT-DCR case had a stricture in the upper canaliculus, and both cases were free of symptoms during follow-up (28 and 38 months).

**Figure 3:**
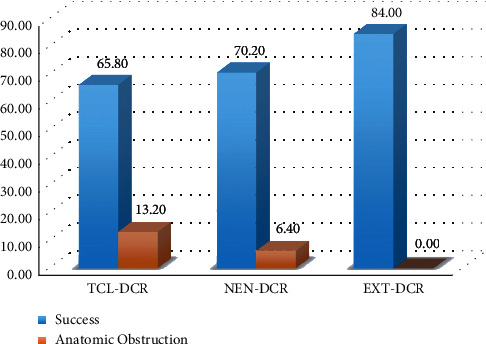
Three different surgical techniques' outcomes.

The success rate was 65.8% in TCL-DCR, 70.2% in NEN-DCR, and 84.0% in EXT-DCR groups. The difference between TCL-DCR and EXT-DCR was statistically significant (TCL-DCR vs. EXT-DCR, *p* < 0.05; TCL-DCR vs. NEN-DCR, *p*=0.66; and EXT-DCR vs. NEN-DCR, *p*=0.11). During follow-up, 13.2% of TCL-DCR and 6.4% of NEN-DCR cases treated for functional epiphora developed a real anatomic obstruction of the lacrimal system at the postsac level that was diagnosed with probing and irrigation. By contrast, all EXT-DCR cases were patent on lacrimal irrigation (*p*=0.04) (Figure 3). The time of failure after surgery was later in the EXT-DCR group (13.3 months) compared with the NEN-DCR (6.9 months) and TCL-DCR (9.5 months) groups (*p*=0.26) (Table 1).

## 4. Discussion

It is already well known that the success rate of DCR in functional epiphora is lower than in primary acquired nasolacrimal duct obstruction (PANDO) [11–13]. Any type of DCR may potentially disrupt and weaken the delicate lacrimal pump [21]. Our study was designed to compare the success of three different DCR techniques, assuming that the extent of damage may vary depending on the surgical technique. All DCRs were combined with bicanalicular silicone intubation to address subtle presac, intrasac, and postsac problems simultaneously [14]. This is the first study in the literature to report the results of TCL-DCR in functional epiphora and compare it with NEN-DCR and EXT-DCR.

**Figure 4:**
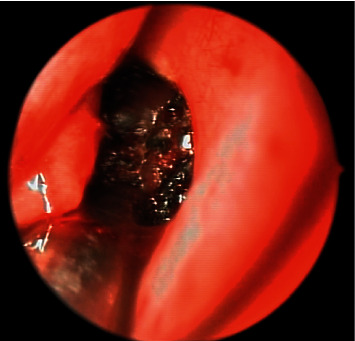
The laser beam causing charring of the nasal mucosa and lacrimal sac during TCL-DCR.

Regarding the success rate of TDL-DCR in PANDO, published results range from 34% to 95.2% in medical literature [22–26]. In a previous study conducted by Kaynak, the long-term functional success of TCL-DCR in PANDO using the same surgical technique by the same surgeons as in this study (PK, CO) was 60.3% after a 2-year follow-up [15]. The low success rate in TCL-DCR was attributed to the thermal damage caused by diode laser on lacrimal and nasal mucosa (Figure 4), together with the absence of flaps promoting excessive secondary wound healing. TCL-DCR with the same surgical technique in functional epiphora cases provided a very similar result in our study (65.8%).

There have been a few studies that look at the thermal effect of 980 nm diode laser on mucosa. Romanos et al. investigated the histological changes in oral mucosa of 18 healthy rabbits following laser-patterned microcoagulation with a 980 nm diode laser to initiate and enhance gingival and oral mucosal tissue regeneration [27]. They came to the conclusion that “laser-patterned microcoagulation treatment with a 980 nm diode laser is a promising method for treating degenerative diseases of the oral soft tissues,” such as gingivitis and gingival recession. Loevschall et al. investigated the effect of low-level diode laser irradiation (LLL) on human oral mucosa fibroblasts in vitro and discovered an increase in DNA synthesis in cultured human oral fibroblasts following LLL exposure [28]. Based on these findings, it is possible that the high amount of laser energy used to create a large ostium increased fibroblastic activity and wound healing, contributing to the relatively low success rate.

Another notable result of this study is that none of EXT-DCRs had an anatomic block after surgery, while 13.2% of TCL-DCR and 6.4% of NEN-DCR cases had a complete block at postsac level confirmed by probing and irrigation. This finding may demonstrate the importance of mucosal flaps for better rhinostomy healing in EXT-DCR and the excessive wound healing related to thermal damage in TCL-DCR. It is known that the toxic tear lake related to ocular surface inflammation may lead to scarring of the lacrimal epithelium and play a vital role in etiopathogenesis of functional epiphora [7, 29, 30]. Given that the EXT-DCR cases had a later failure (13.3 months vs. 6.9 and 9.5 months) and no complete anatomic block of rhinostomy, we can assume that these failures might be related to this chronic inflammation in functional epiphora patients, rather than extensive and irregular healing of rhinostomy after the surgery. Dacryocystorhinostomy failures related to wound healing are mostly expected to happen within the first 12 months after surgery [31, 32]. In our study, each study group had a mean follow-up time longer than 12 months. Therefore, we can assume that the differences in follow-up times between the groups would not change our conclusion about the final results.

The idea of comparing the success of external and endonasal DCR techniques in functional epiphora has been addressed before [33, 34]. One study reported EXT-DCR as being more successful than endoscopic endonasal DCR (EE-DCR) (100% vs. 81%), but another showed an opposite result (EE-DCR 81.3% vs. EXT-DCR 53.9%). The authors suggested that the disruption of the orbicularis muscle and medial canthal tendon in EXT-DCR might harm the lacrimal pump action. Both studies had six months of follow-up, which was shorter than in our study (39.7 months). We found a higher success rate with EXT-DCR compared with TCL-DCR and NEN-DCR (84.0% vs. 65.8% and 70.2%), and the difference between TCL-DCR and EXT-DCR was statistically significant (*p* < 0.05).

In a study comparing lacrimal pump action between EE-DCR and EXT-DCR cases, the signal intensity change at the rhinostomy site after blinking was analyzed on magnetic resonance imaging scans, and a significantly higher intensity was found in the EE-DCR group [35]. This finding was interpreted as better preservation of lacrimal pump action after EE-DCR compared with EXT-DCR. However, in another study, Ciftci et al. emphasized that the meticulous repair of the lacrimal diaphragm layer-by-layer in EXT-DCR helped to preserve lacrimal pump action by reconstructing the attachments of the medial canthal tendon and orbicularis muscle to the lacrimal sac wall [36]. Similarly, in our EXT-DCR procedures, the lacrimal diaphragm was minimally impaired by making a smaller skin incision, not detaching the medial canthal ligament, meticulously repairing the periosteum, and reattaching the orbicularis muscle to the anterior flaps using separate sutures.

NEN-DCR has some advantages over EXT-DCR and TCL-DCR. Dolman stated that the setup for NEN-DCR was simple and less expensive than other techniques involving endoscopes and lasers and that both EXT-DCR and NEN-DCR had a similar success rate in PANDO cases [37]. Additionally, working in the nasal cavity without an endoscope gives the surgeon a larger space for manipulation, and surgical loupes provide enough magnification to view the surgical field. With the absence of thermal damage leading to mucosal scarring and failure, we believe that NEN-DCR can be an alternative for patients concerned about the skin incision in EXT-DCR.

The difference in mean age between the study groups is one of our study's limitations. Despite the fact that older patients are expected to heal at a slower and less aggressive rate, statistical analysis revealed no effect of age on success in our study groups. The main difference between the three study groups in terms of the surgical technique was that MMC was only used as an adjunct in the TCL-DCR group. Mitomycin-C is an antimetabolite and antifibrotic agent that has been shown to improve the success rate of DCR surgery [38]. Nonetheless, in this study, the TCL-DCR group had the lowest success rate. We can assume that the success of TCL-DCR would have been even lower in the absence of MMC, but this does not change our final result. In previous studies, the sensitivity of DCG and FDDT in diagnosing functional epiphora was reported to be similar, and FDDT was considered a simple, reliable, and specific test for identifying PANDO [39, 40]. As a result, despite the fact that different test modalities (DSG or FDDT) were used in this study to evaluate preoperative lacrimal function, we believe that both tests were equally efficient and accurate in diagnosing functional epiphora.

## 5. Conclusion

For the treatment of functional epiphora, the EXT-DCR group had a higher success rate than the NEN-DCR and TCL-DCR groups. This finding suggests that, despite the disruption of the orbicularis muscle, the small skin incision and meticulous repair of the lacrimal diaphragm in EXT-DCR prevented a loss of lacrimal pump action. Furthermore, the absence of an iatrogenic anatomic block in the EXT-DCR group indicates that the mucosal flap anastomosis is critical in increasing the survival of a surgically created ostium. Thermal damage to nasal and lacrimal mucosa caused by diode laser in TCL-DCR may be a key factor responsible for increased wound healing, surgical failure, and iatrogenic total lacrimal obstruction. NEN-DCR, a minimally invasive and simple procedure with a lower success rate, may be a reasonable alternative to EXT-DCR for patients concerned about the appearance of a skin scar.

## Figures and Tables

**Table 1:** Comparison of three distinct groups in terms of demographic and clinical data.

	Demographic and clinical data				
	TCL-DCR	NEN-DCR	EXT-DCR	Total	*P* value
Number of eyes	38	47	50	135	N/A
Gender (F/M)	18/20	34/13	34/16	86/49	0.04
Age (min–max)	49.3 (19–77)	64.1 (37–89)	56.5 (23–82)	57.1 (19–89)	<0.01
Follow-up time in months (min–max)	25.9 (8–85)	44.2 (11–72)	45.9 (6–97)	39.7 (6–97)	<0.01
Time of stent removal in months (min–max)	2.6 (1–5)	2.2 (0–6)	2.3 (0–7)	2.3 (0–7)	0.40
Time of failure in months (min–max)	9.5 (3–46)	6.9 (0–15)	13.3 (4–24)	9.3 (0–46)	0.26

## Data Availability

The clinical data used to support the findings of this study are restricted by the ethics committee of Istanbul University, Istanbul Faculty of Medicine, in order to protect patient privacy. The SPSS data used to support the findings of this study are available from the corresponding author upon reasonable request.

## Supplementary Materials

Supplementary Materials The brief video demonstrates the critical steps of a successful TCL-DCR surgery with MMC application.
