# Supplementary material for: A Comparison of Transcanalicular, Endonasal, and External Dacryocystorhinostomy in Functional Epiphora: A Minimum Two-Year Follow-Up Study
Source: J Ophthalmol. 2022 Mar 23;2022:3996854. doi: 10.1155/2022/3996854 (PMC8967575; doi:10.1155/2022/3996854)
Supplement: Supplementary Materials — The brief video demonstrates the critical steps of a successful TCL-DCR surgery with MMC application. [file 3996854.f1.docx]

Supplementary:

Since the supplementary video file cannot be submitted under the supplementary section, it has been updated under the figures and tables section. Please use them for further processing.
